# Sex-related disparities in outcomes of cholangiocarcinoma patients in treatment trials

**DOI:** 10.3389/fonc.2022.963753

**Published:** 2022-08-11

**Authors:** Matthew Ledenko, Samuel O. Antwi, Shiho Arima, Julia Driscoll, Junji Furuse, Heinz-Josef Klümpen, Finn Ole Larsen, David K. Lau, Annett Maderer, Alice Markussen, Markus Moehler, Lynn E. Nooijen, Walid L. Shaib, Niall C. Tebbutt, Thierry André, Makoto Ueno, Rachel Woodford, Changhoon Yoo, Mark M. Zalupski, Tushar Patel

**Affiliations:** ^1^ Department of Transplantation, Division of Gastroenterology and Hepatology, Mayo Clinic, Jacksonville, FL, United States; ^2^ Department of Quantitative Health Sciences, Mayo Clinic, Jacksonville, FL, United States; ^3^ Department of Digestive and Lifestyle Diseases, Kagoshima University Graduate School of Medical and Dental Sciences, Kagoshima, Japan; ^4^ Department of Medical Oncology, Kyorin University Faculty of Medicine, Tokyo, Japan; ^5^ Department of Medical Oncology, Cancer Center Amsterdam, Amsterdam University Medical Center (UMC), Amsterdam, Netherlands; ^6^ Department of Oncology, Copenhagen University Hospital, Herlev and Gentofte Hospital, Herlev, Denmark; ^7^ Oncogenic Transcription Laboratory, Olivia Newton-John Cancer and Research Institute, Melbourne, VIC, Australia; ^8^ School of Cancer Medicine, La Trobe University, Melbourne, VIC, Australia; ^9^ First Department of Medicine, University Medical Center of the Johannes-Gutenberg University, Mainz, Germany; ^10^ Research Center for Immunotherapy (FZI), University Medical Center, Johannes Gutenberg-University, Mainz, Germany; ^11^ Department of Surgery, Cancer Center Amsterdam, Amsterdam UMC, Amsterdam, Netherlands; ^12^ Department of Hematology and Medical Oncology, Winship Cancer Institute, Emory University, Atlanta, GA, United States; ^13^ Department of Medical Oncology, Olivia Newton-John Cancer Centre at Austin Health, Heidelberg, VIC, Australia; ^14^ Sorbonne University and Department of Medical Oncology, Hôpital Saint-Antoine, AP-HP, Paris, France; ^15^ Department of Gastroenterology, Hepatobiliary and Pancreatic Medical Oncology Division, Kanagawa Cancer Center, Kanagawa, Japan; ^16^ National Health and Medical Research Council Clinical Trials Centre (NHMRC CTC), Medical Foundation Building, University of Sydney, Camperdown, NSW, Australia; ^17^ Department of Oncology, Asan Medical Center, University of Ulsan College of Medicine, Seoul, South Korea; ^18^ Department of Medicine, Division of Hematology and Oncology, University of Michigan, Ann Arbor, MI, United States

**Keywords:** Biliary cancers, chemotherapy, gemcitabine, therapeutic response, survival

## Introduction

Cholangiocarcinoma (CCA) is a heterogeneous group of malignancies arising from the biliary tract. Three main types of CCA are recognized: intrahepatic, distal CCA, and perihilar CCA. These cancers have poor prognosis because they often progress without symptoms and are typically diagnosed at advanced stage. Surgical interventions with microscopically negative resection margins (R0) can be curative but are not an option for nearly two-thirds of patients who present with unresectable disease (1). Moreover, the intrinsic and acquired chemoresistance of these tumors limits responses to therapy (2–4).

Understanding the determinants of chemoresistance in these cancers can improve our knowledge of disease pathogenesis, progression, and help improve therapeutic response. In general, mortality associated with primary liver cancers is higher in males than females, which could be due to sex-dependent biological effects, intrinsic differences in the natural history of tumor progression, or sex-related alterations in therapeutic responses (5). Further, males and females could respond differently to treatment due to sex-dependent variation in metabolism, drug pharmacokinetic or pharmacodynamic activities (6), but the impact of sex on therapeutic response in CCA is not known. In most reports of treatment trials, outcome data from study participants are combined for males and females, which may mask sex-based variation in treatment responses. A glaring gap in treatment trials data is that most trials do not perform randomization by sex. To evaluate the possibility that therapeutic responses in CCA differ by biological sex, we conducted a systematic review of the effect of sex on the outcomes of treatments in reported therapeutic trials.

## Materials and methods

### Search strategy

A systematic search strategy for identifying studies was developed in accordance with the PRISMA guidelines. The approach was prospectively registered on PROSPERO on 19/09/2021 (Registration number CRD42021273679). The strategy was applied to the following databases/registers: PubMed.gov, Clinicaltrials.gov, Cochrane Central Register of Controlled Trials, EMBASE via Ovid, the World Health Organization’s International Clinical Trial Registry Platform, and Google Scholar. Search inputs used combinations of medical subject headings (MeSH) and Boolean operator searching to find both non-randomized and randomized trials. Combinations of the search terms were used, such as: “cholangiocarcinoma” [MeSH], “biliary tract neoplasms” [MeSH], “cholangiocarcinoma” [Tiab], OR “biliary cancer”, AND “Overall survival”. The searches performed, terms used and results for each database and registry are described in **Supplementary Tables S1, S2**.

### Inclusion and exclusion criteria

Inclusion criteria were defined *a priori* as any study that had enrolled a minimum of four patients with CCA, with all patients aged 18 years or more and which involved a therapeutic treatment arm. Exclusion criteria included adjuvant therapeutic trials that involved the concomitant use of surgery, radiation, immunotherapy, or other treatments besides drug therapy, trials that involved direct tumoral infusion (e.g., intrahepatic administration) or trials that explicitly focused on palliative care.

### Database search

Data from all database or registry searches were extracted into excel spreadsheets and included the following information: first author, year, identification number (e.g., PubMed ID, NCT-ID), title, and a link to the full text. Duplicates within datasets were identified and removed through manual and automated cross comparing. Two independent reviewers subsequently reviewed each record for retrieval. Discrepancies in agreement were very few and each of them was discussed together to arrive at consensus. Studies that were accepted by both reviewers were reviewed for specific sex-related outcomes for data extraction. The corresponding author for each study was contacted by email, with a follow-up request for those who did not respond after two weeks. The email included a standardized data request form to the listed corresponding author for each eligible study.

### Data collection and analysis

For each study, data for mean age, number of patients, body mass index (BMI), socioeconomic status (SES), history of prior resections and the median and range of overall survival (OS) and progression-free survival (PFS) were requested for males and females separately. All data were subsequently logged and used in the analysis. None of the studies were able to provide SES data. Although not specifically requested, data from studies that provided 95% confidence interval (CI) data was accepted. Sex-specific survival data visualization was performed using bar graphs, scatter plots, and bubble plots. Risk of bias was not assessed due to the lack of sex-based randomization. Each of the studies included had verified in their published reports that written informed consent was obtained from study participants, and that the study protocols conformed to the ethical guidelines of the 1975 Helsinki Declaration as reflected in *a priori* approvals by their respective institutional human subjects research committees.

## Results

### Selection of studies

**Figure 1:**
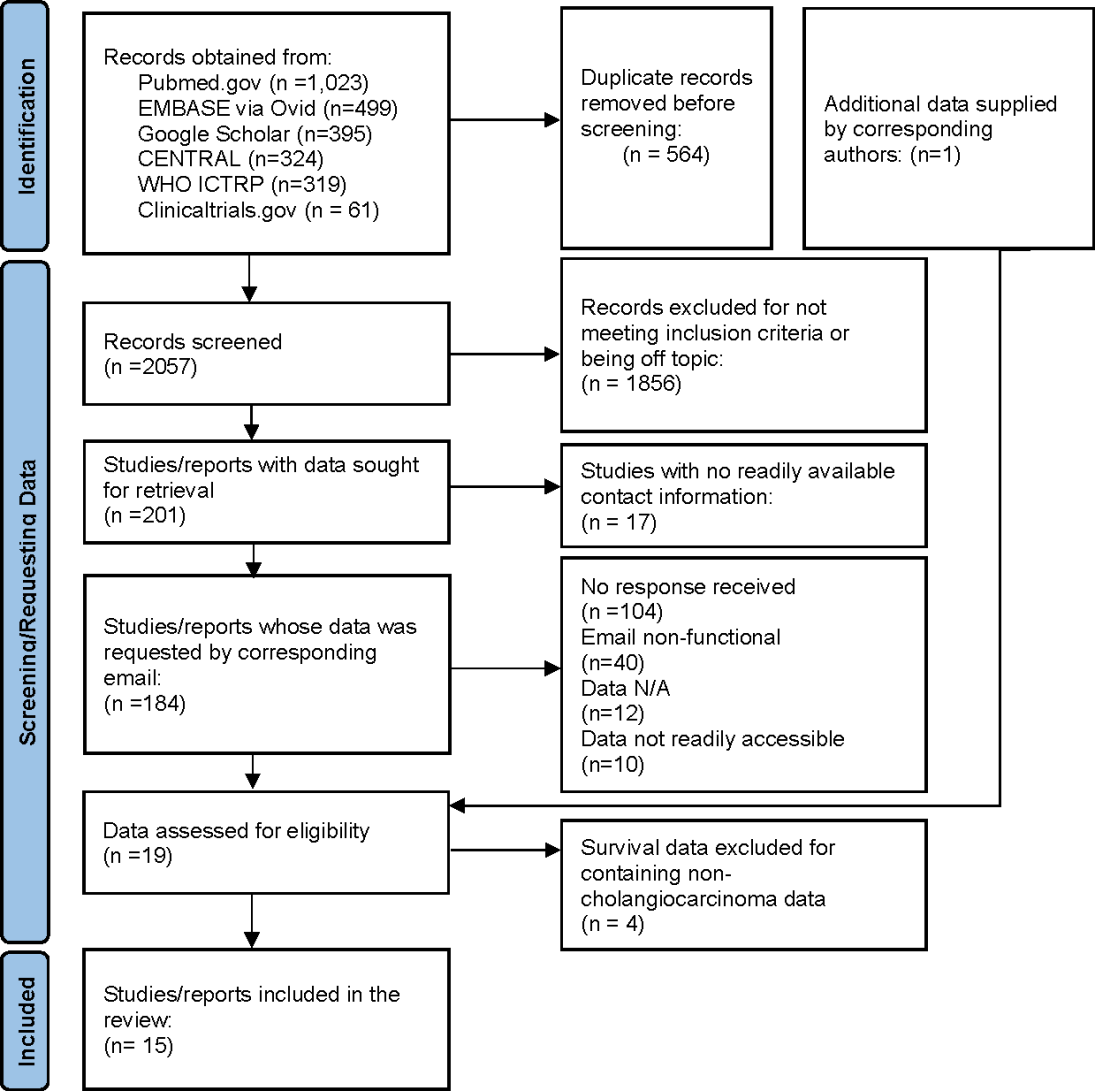
Identification of studies. Flowchart of the systematic search to identify studies and selection of studies included in the analysis.

The results of the screening and literature searches are outlined in the flow chart (**Figure 1**). A total of 2,621 records were obtained from all searches, of which 564 records were removed for being duplicates. The remaining 2,057 records were screened for eligibility by two independent reviewers. Of these, 201 were identified as being appropriate for this review by consensus of both reviewers and were selected. Contact information was identified for 184 studies, and the corresponding or senior authors were contacted by e-mail. Of these, 104 did not respond to two separate requests, and 40 had non-functional emails. Amongst those who responded,12 indicated that the requested data was not collected or was missing, 10 responded that they were unable to provide the data or that it was not readily available, 4 met the criteria but provided data for participants that did not meet criteria and 15 provided CCA-specific data for inclusion in this analysis. Each of the 15 studies was further reviewed for appropriateness. Each met the inclusion criteria and the datasets provided were deemed to be suitable for analysis and inclusion in this review.

### Identification of treatment groups

The fifteen studies reported data from 587 patients with CCA of which 309 were males and 278 were females. Characteristics of these studies are listed in **Table 1** (7–21). The trials were performed between 2008 and 2021, and the recruitment sites spanned multiple global locations including North America, Europe, Asia, South America, and Australia. Treatment allocation was randomized in 4 trials and was non-randomized in 11 trials. However, randomization was not performed by sex in any of the studies.

**Table 1:** Characteristics of studies included in the review.

Study	Allocation	Group assignment	Study duration	Country
André 2008 (7)	Non-randomized	Single	2003-2005	France, Germany, Austria, Chile, UK
Lassen 2010 (8)	Non-randomized	Single	2004-2008	Denmark
Moehler 2014 (9)	Randomized	Parallel	2008-2010	Germany
Ole Larsen 2015 (10)	Non-randomized	Single	2011-2016	Denmark
Arima 2017 (11)	Non-randomized	Single	2008-2011	Japan
Lau 2018 (12)	Non-randomized	Single	2009-2011	Australia
Davis 2018 (13)	Non-randomized	Single	2011-2016	United States
Mazzaferro 2019 (14)	Non-randomized	Single	2012-2018	United States, Italy
Moehler 2019 (15)	Non-randomized	Single	2012-2016	Germany
Belkouz 2020 (16)	Non-randomized	Single	2016-2018	Netherlands
Markussen 2020 (17)	Randomized	Parallel	2014-2017	Denmark
Ueno 2021 (18)	Randomized	Parallel	2018-2019	China
Zhang 2021 (19)	Non-randomized	Single	2017-2018	China
Yoo 2021 (20)	Randomized	Parallel	2018-2020	South Korea
Woodford 2021 (21)	Non-randomized	Single	2015-2016	Australia

Of the fifteen studies, four had parallel assignments (i.e., eight treatment groups) whereas 11 had a single group assessment. Therefore, there were 19 separate treatment groups for analysis. Sixteen of the 19 patient groups had a female-to-male mean age ratio between 0.9 and 1.1, indicating similar spread of age across groups. The survival data for males and females in each of these 19 groups is presented in **Table 2**. All except one of the treatment groups (8) included patients with unresectable or metastatic disease. Sixteen of the 19 treatment groups included patients with either intrahepatic or extrahepatic CCA, while one group included only intrahepatic CCA. Tumor location was not specified in two of the groups (**Supplementary Table S3**).

**Table 2:** Survival data of included studies.

Study	Drug(s)	Sex	CCA Patients	Mean Age (years)	OS (months)			PFS (months)		
					Median	Range		Median	Range	
André 2008 (7)	Gemcitabine + Oxaliplatin	Male	18	58.0	6.9	3.0-27.7		N/A	N/A	
		Female	19	58.4	9.1	1.4-29.4		N/A	N/A	
Lassen 2010 (8)	GemCapOx**	Male	16	58.8	10.2	2.1-23.4		7.4	0.2-23.4	
		Female	25	63.1	10.3	0.4-23.4		6.7	0.4-15.7	
Moehler 2014 (9)	Gemcitabine + Sorafenib	Male	15	61.5	10.3	0.3-23.6		3.2	0.0-16.4	
		Female	28	62.8	13.3	0.9-27.5		1.8	0.9-19.1	
Moehler 2014 (9)	Gemcitabine + Placebo	Male	21	62.5	6.7	0.3-27.1		4.9	0.0-16.8	
		Female	20	64.3	10.2	1.1-23.5		3.4	1.1-20.7	
Ole Larsen 2015 (10)	GemCapOx**	Male	22	N/A	13.3	11.3-15.2		7.7	4.6-10.8	
		Female	25	N/A	10.5	9.1-12.0		7.5	5.8-9.2	
Arima 2017 (11)	Gemcitabine + S-1	Male	16	64.0	12.3	3.7-38.3		4.4	1.1-15.5	
		Female	10	65.6	20.9	1.8-42.0		10.5	1.7-26.1	
Davis 2018 (13)	GemCis + 5-FU**	Male	4	70.2	16.5	2.1-36.6		6.7	1.6-13.4	
		Female	4	50.3	15.5	7.9-26.3		7.6	3.0-15.6	
Lau 2018 (12)	Everolimus	Male	5	63.8	9.5	1.7-25.4		12.7	1.3-19.5	
		Female	7	56.8	14.4	1.1-20.5		10.5	1.1-17.0	
Mazzaferro 2019 (14)	Derazantinib	Male	2	45.6	8.7	1.6-15.8		4.8	1.6-8.5	
		Female	11	56.8	18.7	5.1-27.3		10.4	3.4-16.5	
Moehler 2019 (15)	GemCis + Afatinib**	Male	5*	64.6	5.4	2.6-9.4		6.0	2.1-7.7	
		Female	2	56.0	11.1	6.9-18.5		6.9	1.5-11.0	
Markussen 2020 (17)	GemCis**	Male	19	62.2	15.2	4.0-32.3		8.4	2.1-30.1	
		Female	17	63.7	8.6	0.8-31.2		5.7	0.6-24.5	
Markussen 2020 (17)	GemCapOx**	Male	19	60.2	9.5	0.2-38.6		7.3	0.2-24.8	
		Female	13	63.7	10.5	1.0-24.4		7.7	0.9-22.8	
Belkouz 2020 (16)	FOLFIRINOX**	Male	15	57.9	7.6	3.0-27.7		4.5	1.9-19.4	
		Female	8	62.6	21.5	4.5-25.7		5.8	1.8-17.7	
Woodford 2021 (21)	Capecitabine + Nab-paclitaxel	Male	7	63.8	12.5	2.8-24.6		9.1	1.3-14.8	
		Female	3	63.1	26.2	4.3-29.6		3.5	3.5-3.5	
Ueno 2021 (18)	S-1 + Placebo	Male	23	66.7	7.6	0.8-14.9		3.0	0.0-14.8	
		Female	13	64.7	7.9	3.9-13.9		3.0	1.4-13.0	
Ueno 2021 (18)	S-1 + Resminostat	Male	16	61.6	7.2	2.0-16.7		2.9	1.1-11.2	
		Female	15	59.2	7.6	1.7-12.3		3.0	1.2-9.2	
Zhang 2021 (19)	Apatinib	Male	13	53.6	5.7	0.9-20.4		3.1	0.7-8.0	
		Female	11	56.2	10.2	2.5-26.8		2.4	1.2-8.2	
Yoo 2021 (20)	5-FU/Leucovorin**	Male	38	64.7	5.4	0.5-18.8		1.8	1.8-14.5	
		Female	26	62.5	6.7	0.5-17.5		1.5	0.3-6.9	
Yoo 2021 (20)	nal-Irinotecan + 5-FU/Leucovorin**	Male	36	62.8	7.7	1.5-14.3		2.8	0.6-13.5	
		Female	21	64.0	8.6	2.0-12.9		3.7	1.0-16.3	

*Five male patients were used in the male OS calculations and three male patients were used in the PFS calculations.

**5-FU, 5-flourouracil; CCA, cholangiocarcinoma; FOLFIRINOX, leucovorin + 5-FU + irinotecan + oxaliplatin; GemCapOx, gemcitabine + capecitabine + oxaliplatin; Gemcis, gemcitabine + cisplatin; N/A, not available; OS, overall survival; PFS, progression-free survival.

### Differences in overall survival between males and females

**Figure 2:**
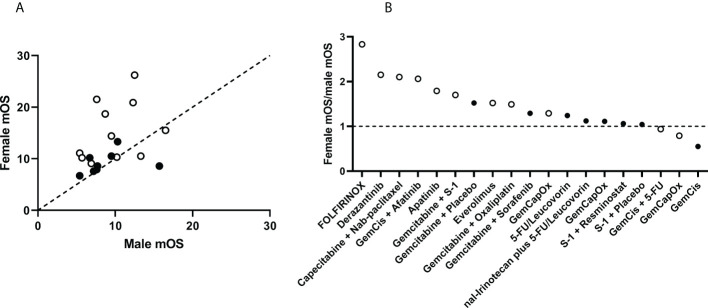
Comparison of median overall survival (OS) in female and male participants in treatment trials of cholangiocarcinoma. **(A)** Data were obtained from participants in 19 treatment groups from 15 studies. Treatment groups above the dotted line of equivalence have a higher overall survival in females compared with males. **(B)** The ratio of median OS in females to median OS in males is plotted for each treatment group. Treatments with higher median OS in females are plotted above the dotted line, with ratio >1. Randomized of treatment was performed in studies represented by solid dots, non-randomized studies as open dots.

The analysis indicated that females have a tendency towards a higher overall survival than males enrolled in treatment trials of CCA (**Figure 2**). There were several treatment groups that had higher total female overall survival rates, such as derazantinib, capecitabine + nab-paclitaxel, and gemcitabine + cisplatin (GemCis) + afatinib. The differences were most noticeable for FOLFIRINOX treatment in which the female-to-male ratio of median OS was 2.83. The median OS was higher in females in most of the other treatment regimens, with a female-to-male ratio in overall survival above 1.1 in 14 treatment groups. In the rest of the treatment groups, three had ratios ranging from 1.1 to 0.9, and only two had a ratio below 0.9.

### Differences in progression-free survival between males and females

**Figure 3:**
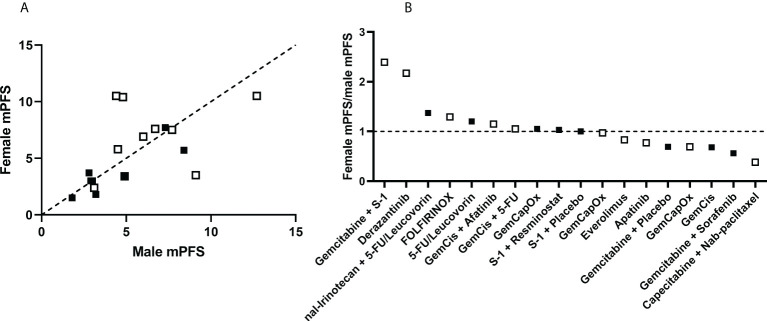
Comparison of disease progression in female and male participants in treatment trials of cholangiocarcinoma. **(A)** Data were obtained from participants in 19 treatment groups from 15 studies. Treatment groups above the dotted line of equivalence have a higher median progression free survival (mPFS) in females compared with males. **(B)** The ratio of mPFS in females to mPFS in male is plotted for each treatment group. Treatments with higher mPFS in females are plotted above the dotted line, with ratio >1. Randomized of treatment was performed in studies represented by solid dots, non-randomized studies as open dots.

**Figure 4:**
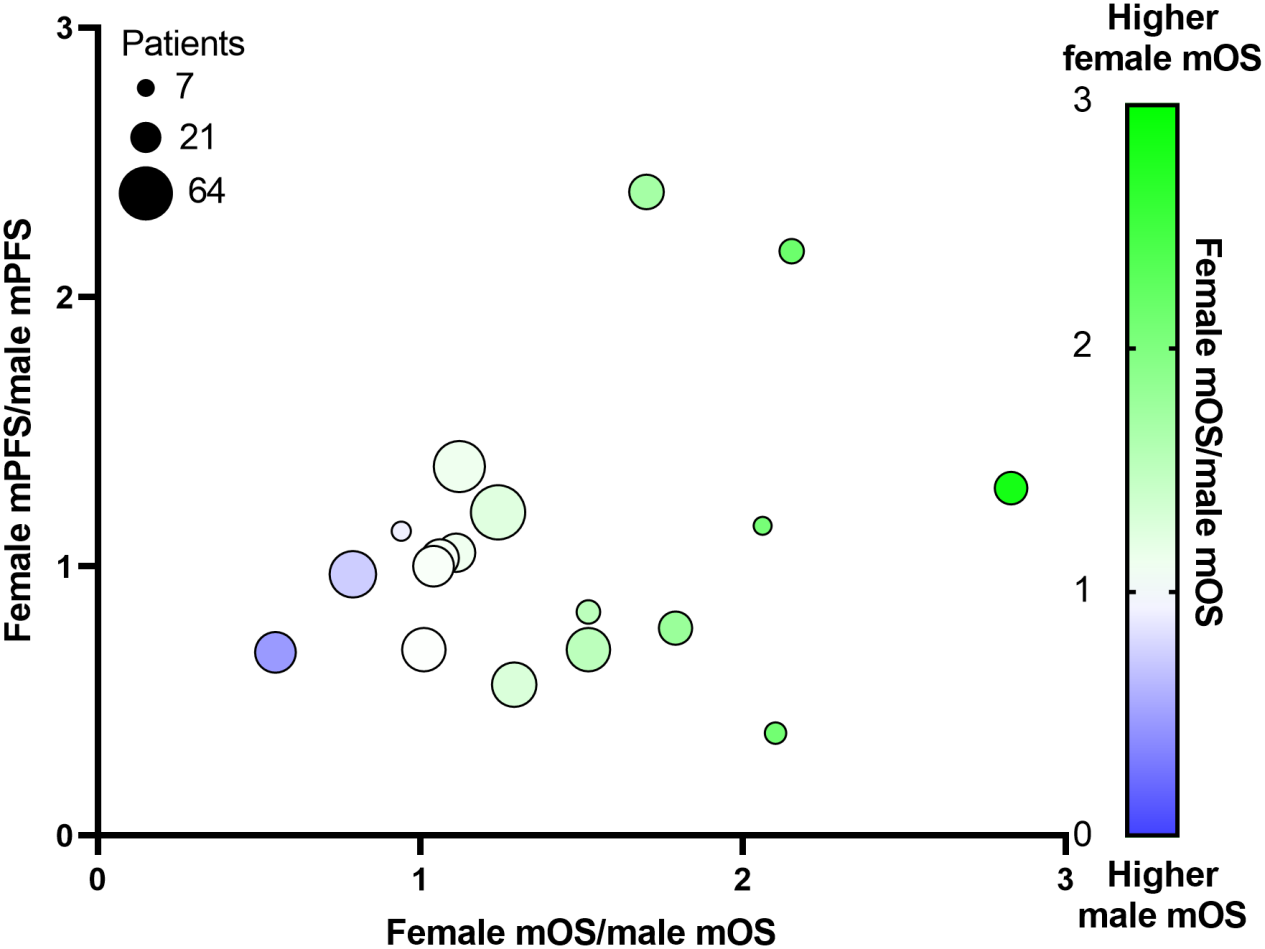
Relationship between study size, median overall survival (mOS) and median progression-free survival (mPFS) in males and female participants of treatment trials in cholangiocarcinoma. The female-to-male ratio of mOS is plotted against the female-to-male ratio of mPFS. The size of the plot is proportional to the number of participants in the treatment group.

The data on median PFS in these studies differed from those for overall survival. While differences in median PFS data were observed, these had an equal distribution above and below the equivalence line indicative of varying effects between the sexes (**Figure 3**). Seven treatment groups had median PFS ratios above 1.1, five had ratios from 1.1-0.9, and seven had ratios below 0.9. Notably, more extreme differences were observed in studies with a smaller sample size, whereas larger studies showed more moderate differences (**Figure 4**). Of note, higher survival rates were noted in males in only one treatment regimen with GemCis, with a female-to-male ratio < 1.0 for both median OS and median PFS.

### Differences in survival between males and females for gemcitabine-based regimens

Understanding the effects of biological sex on responses to gemcitabine-based regimens is clinically relevant but also of high importance. Both before and more so after the landmark ABC-02 study established GemCis as the standard of care, gemcitabine has been widely used for the treatment of CCA. To evaluate this further, we examined the potential sex-related differences in survival outcomes across treatment regimens containing gemcitabine. Survival data was compiled from 10 treatment groups which included gemcitabine (**Table 2**, **Supplementary Figure S1**). Outcomes across treatment groups were heterogeneous, with exception to the gemcitabine, capecitabine, and oxaliplatin (GemCapOx) treatment groups, which had a median OS within 0.2 months of each other. Out of the ten treatment groups, median OS was higher in females in six groups, with the greatest effect noted for treatment with gemcitabine + S-1. For the other four groups, median OS was equivalent (e.g., for GemCapOx) or better in males (e.g. GemCis or gemcitabine + oxaliplatin). While dosage schedules and the number of study participants were comparable across all three GemCapOx treatment groups, an improved OS in males was observed in only one of the three studies (**Supplementary Table 3**).

As noted above, the biological sex-associated impact on disease-free progression was also notably distinct from those for overall survival. Similar sex-based effects in progression-free and overall survival data were noted for a few treatments (**Table 2**, **Supplementary Figure S1B**). For others, sex-based effects varied between disease progression and overall survival. For instance, while median OS was higher in females treated with gemcitabine + sorafenib or gemcitabine + placebo, these were different for median PFS in the same studies. Of note the median PFS was lower in both sexes with sorafenib treatment compared to placebo and suggesting sorafenib might be similarly intolerable for both sexes.

### Differences in survival between males and females for non-gemcitabine-based regimens

We next analyzed outcomes in treatments which did not contain gemcitabine (**Table 2**, **Supplementary Figure S2**). Out of nine treatment groups, median OS was higher in females than in males in seven studies but similar in only two studies. In a randomized trial of 5-FU/leucovorin or 5-FU/leucovorin with nal-irinotecan, overall survival in females exceeded that of males by 0.9 and 1.3 months respectively. However, the same was not true for a randomized study of S-1 + placebo and S-1 + resminostat in which female median OS was only slightly higher (0.3 months) in males indicating then absence of a significant relationship between sex and outcomes. In this trial, male-to-female ratios for median PFS were like those for OS.

While a higher median PFS was observed in females treated with 5-FU/nal-irinotecan (**Table 2**, **Supplementary Figure S2B**), the data was more varied in studies with other treatments, with four having higher median PFS in males, and two being similar between sexes. Discordant effects in median PFS and median OS between males and females were noted for other treatment groups, such as with everolimus and capecitabine + nab-paclitaxel.

While gemcitabine has remained a critical part of CCA treatment regimens, there are many novel therapies that have demonstrated verifiable and significant promise. Pemigatinib and ivosidenib, both of which were approved by the Food and Drug Administration in 2020-2021 for selective use in a subgroup of CCA with FGFR2 alterations are evolving options for the treatment of CCA in certain settings. As newer therapeutic regimens undergo testing, it will be pertinent to understand any sex-related effects on survival outcomes.

## Discussion

A major gap in evolving treatments for CCA is an understanding of the effect of biological sex on outcomes of treatment. As treatment options for CCA evolve, this knowledge will be essential not only for the design and conduct of the most appropriate clinical trial designs, but also guide towards appropriate selection of treatment options for patients with CCA. In this analysis, a distinctive sex-related difference in overall survival was observed in an analysis of responses to treatment from participants in completed trials of CCA. The data herein was comprised from 587 patients of which 47% were females. Females enrolled in treatment trials of CCA had a higher OS than males in two-thirds of all treatment groups. In contrast, disease progression, as determined by the median PFS across all treatment groups had similar outcomes between the sexes.

The mortality from CCA is lower among females compared with males in recent data from the United States, with a risk ratio of 0.78 (95% CI 0.77–0.79) (22). Thus, the impact of sex on treatment outcomes is important, and especially so given the increasing CCA incidence and mortality from CCA observed in the United States and many other countries (23). There are several factors that can contribute to sex-dependent differences, such as sex-dependent effects on the underlying liver disease, pathobiological processes or concomitant comorbidities. For example fibrogenesis can occur in a sex-dependent manner in non-alcoholic fatty liver disease. Etiological factors, such as cirrhosis, viral hepatitis, presence and extent of sarcopenia and smoking can also contribute (24). In particular, smoking can impact both first line and neoadjuvant chemotherapy responses in some cancers (25, 26). Variable responses in different types of tumors to therapeutic interventions such as surgical resection and chemotherapy could also contribute to disparities in survival. Most of the studies reported herein aggregated intrahepatic and extrahepatic CCA data precluding tumor type-specific analyses. Complete data on tumor type, underlying liver disease or etiology, or smoking history were not available for the studies reported but warrant specific assessment in future clinical trials. While sex can affect drug metabolism, responses, and resistance, the effect of biological sex on treatment responses has not previously been widely recognized as a potential contributor to these differences. Differences in epigenetic changes between cis and trans participants raises the complexities involved and need for consideration for trans gender identities as well (27).

This study has several limitations. None of the studies involved randomization by sex. Moreover, patient-based characteristics that could potentially confound the observations, such as BMI or SES could not be accounted for. Thus, sex-dependent differences in recruitment, disease severity, BMI, SES may all have contributed. While this may be less likely given the effects were observed across multiple diverse trials, future trials with sex-based randomization and comparability across groups would be necessary to completely evaluate these possibilities. Many of the studies had small sample sizes. While low numbers of patient enrollments are characteristic of many studies of CCA, which is a rare disease, a smaller sample size can skew results of treatment effects. The absence of sex-specific reporting of survival outcomes in reported trials was particularly noteworthy and highlights a major limitation. Of the 184 publications initially identified, few if any reported non-aggregated data on survival in females and males. A further challenge with retrospective systematic reviews is the susceptibility to selection bias introduced due to inaccessible or lost data. In this context, sequestration and non-sharing of trial data by individuals or organizations hampers progress and erodes the trust placed by participants who enroll in trials to advance medical knowledge.

Some drugs such as 5-FU, cisplatin, and nab-paclitaxel have sex-dependent effects in different tissues (28). Sex-dependent expression of phase I and phase II drug metabolizing enzymes can result in differential metabolism and clearance, and thereby impact therapeutic efficacy of many drugs (29, 30). Amongst these, the regulation and expression of cytochrome P450 (CYP) gene and enzyme families are of particular importance as these genes are sex-based, and dependent on enhancer of zest homolog 1 and 2 (31). For example, CYP3A4 has higher expression levels in female livers and has been implicated in the biotransformation of more than 50% of all clinically used drugs (32). Differential regulation of CYP gene and enzyme families could thereby contribute to sex differences in outcomes.

Intrinsic or acquired resistance to drugs contributes to the difficulty in treating CCA. Variable effects on gene expression, and regulation of metabolic pathways can all contribute to sex differences in drug resistance. P-glycoprotein serves as an important ATPase transporter protein that can operate as a drug efflux pump for a variety of drugs. Commonly found in hepatocytes and intestinal enterocytes, overexpression of P-gp is linked to multidrug resistance and worse clinical outcomes in cancer (33). A higher hepatic expression of P-gp in men could thus account for the higher overall mortality rates observed in men with CCA compared with women (34). Hormonal differences can further impact on sex-dependent differences. In addition to direct endocrinological effects, the activity of certain P-gp and CYP isoforms can be regulated by progesterone and estrogen levels, respectively (35, 36). However, the relationships in the liver-related CYP and P-gp isoforms are poorly understood.

The results of this analysis raise awareness and a call for action to consider sex-based differences in outcomes while designing future treatment trials for CCA or in the management of CCA. Randomization based on biological sex, and consideration of gender-based dosing may need to be considered where data on sex differences in drug efficacy and metabolism is available.

## Data availability statement

The original contributions presented in the study are included in the article/supplementary material. Further inquiries can be directed to the corresponding author.

## Author contributions

TP and ML contributed equally to the writing of the manuscript; TP oversaw the design and execution of the review; ML performed the systematic searches, data acquisition, and data processing; rest of authors contributed data and/or performed a critical review of the manuscript. All authors contributed to the article and approved the submitted version.

## Funding

Support for this study was provided by the James C and Sarah K Kennedy Deanship and Alfred D. and Audrey M. Petersen Professorship at Mayo Clinic to TP.

## Acknowledgments

We are grateful for the contributions of all participants in the treatment trials, the investigators who provided data including Wei He and Ulrik Lassen, and for members of the Patel Lab for their insights.

## Conflict of interest

The authors declare that the research was conducted in the absence of any commercial or financial relationships that could be construed as a potential conflict of interest.

## Publisher's note

All claims expressed in this article are solely those of the authors and do not necessarily represent those of their affiliated organizations, or those of the publisher, the editors and the reviewers. Any product that may be evaluated in this article, or claim that may be made by its manufacturer, is not guaranteed or endorsed by the publisher.

## Abbreviations

5-FU, 5-fluorouracil; BMI, body mass index; CI, confidence interval; CYP, cytochrome P450; GemCapOx, gemcitabine, capecitabine, oxaliplatin; GemCis, gemcitabine and cisplatin; FOLFIRINOX, 5-flourouracil, leucovorin, irinotecan, oxaliplatin; iCCA, intrahepatic cholangiocarcinoma; mOS, median overall survival; mPFS, median progression-free survival; Nal-Irinotecan, nanoliposomal irinotecan; P-gp, p-glycoprotein; SES, socioeconomic status.
