# Supplementary material for: Sex-related disparities in outcomes of cholangiocarcinoma patients in treatment trials
Source: Front Oncol. 2022 Aug 11;12:963753. doi: 10.3389/fonc.2022.963753 (PMC9404243; doi:10.3389/fonc.2022.963753)
Supplement: Supplementary file 1 [file DataSheet_1.docx]

**Supplementary Information**

**Sex-related disparities in outcomes of cholangiocarcinoma patients**

**in treatment trials**

Matthew Ledenko, Samuel O. Antwi, Shiho Arima, Julia Driscoll, Junji Furuse, Wei He, Heinz-Josef Klümpen, Ulrik Lassen, Finn Ole Larsen, David K. Lau, Annett Maderer, Alice Markussen, Markus Moehler, Lynn E. Nooijen, Walid Shaib, Niall C. Tebbutt, Thierry André, Makoto Ueno, Rachel Woodford, Changhoon Yoo, Mark M Zalupski, Tushar Patel

**Supplementary Appendix**

**Table of contents:**

**Supplementary Table S1:………………………..……………..……………………….….…...........……….p. 2-3**

**Supplementary Table S2:………………………..……………..……………………….….…...........……….p. 4**

**Supplementary Table S3:………………………..……………..……………………….….…...........……….p. 5-7**

**Supplementary Fig. S1:…………………………………………………………………………..………….....p. 8**

**Supplementary Fig. S2:………………………………………….……………………………….………….....p. 9**

**Supplementary Table S1: Terms used for literature searches**

| **Search** | **Source** | **Website** | **Terms** |
| --- | --- | --- | --- |
| **1** | **Pubmed** | **https://pubmed.ncbi.nlm.nih.gov/** | ("Cholangiocarcinoma"[Mesh]) OR "Biliary Tract Neoplasms"[Mesh] OR "Cholangiocarcinoma"[Tiab] AND (randomizedcontrolledtrial[Filter]) AND (1975:2021[pdat])) |
| **2** | **Pubmed** | **https://pubmed.ncbi.nlm.nih.gov/** | (("Cholangiocarcinoma"[Mesh]) OR "Biliary Tract Neoplasms"[Mesh] OR "Cholangiocarcinoma"[Tiab]) NOT "Stent"[Tiab] NOT (randomizedcontrolledtrial[Filter]) AND ((clinicaltrial[Filter]) AND (1969:2021[pdat])) |
| **3** | **Cochrane Central Register of Controlled Trials (CENTRAL)** | **https://www.cochranelibrary.com/central** | #1 MeSH descriptor: [Cholangiocarcinoma] explode all trees  #2 MeSH descriptor: [Biliary Tract Neoplasms] explode all trees  #3 ("Cholangiocarcinoma" OR "Bile Duct Cancer"):ti,ab,kw  #4 (NOT "stent"):ti,ab,kw  #5 ("overall survival")  #6 ("progression free survival")  #7 (#1 OR #2 OR #3) AND #4 AND (#5 OR #6) in Trials |
| **4** | **NIH Database of Clinical Trials** | **https://clinicaltrials.gov/** | Studies with Results \| "Bile duct cancer" OR "Cholangiocarcinoma" \| Adult, Older Adult \| Results first posted from 01/01/1965 to 08/17/2021 |
| **5** | **World Health Organization International Clinical Trial Registry (WHO-ICTRP)** | **https://www.who.int/clinical-trials-registry-platform/the-ictrp-search-portal** | NOT \| "Stent" OR "Anti-PD-1" OR "PD-1" OR "PD1" OR "Pembrolizumab" OR "Nivolumab" OR "Durvalumab" OR "Immunotherapy" OR "Radiation" OR "Photodynamic" OR "Adjuvant" OR "Neoadjuvant" OR "Arterial Infusion" \| AND "Cholangiocarcinoma" OR "Bile Duct Cancer" OR "Bile Tract Cancer" OR "Biliary Cancer" |
| **6** | **Excerpta Medica database (EMBASE)** | **https://www.embase.com/** | [1] bile duct carcinoma/dm, dt, th [Disease Management, Drug Therapy, Therapy]  [2] bile duct cancer/dm, dt, th [Disease Management, Drug Therapy, Therapy]  [3] (Cholangiocarcinoma or "Bile Duct Cancer" or "Biliary Cancer").mp. [mp=title, abstract, heading word, drug trade name, original title, device manufacturer, drug manufacturer, device trade name, keyword, floating subheading word, candidate term word]  [4] phase 3 clinical trial/ or phase 1 clinical trial/ or clinical trial protocol/ or "randomized controlled trial (topic)"/ or clinical trial/ or superiority trial/ or adaptive clinical trial/ or phase 4 clinical trial/ or randomized controlled trial/ or non-inferiority trial/ or controlled clinical trial/ or phase 2 clinical trial/ or "phase 3 clinical trial (topic)"/ or veterinary clinical trial/ or "phase 4 clinical trial (topic)"/ or pragmatic trial/ or "phase 1 clinical trial (topic)"/ or "controlled clinical trial (topic)"/ or "phase 2 clinical trial (topic)"/ or "trial of labor"/ or equivalence trial/  [5] ((1 or 2 or 3) and 4 and ("Overall survival" or "Progression free survival").mp.) not Stent.mp. not Immunotherapy.mp. not stereotactic.mp. not Pembrolizumab.mp. not Nivolumab.mp. not Durvalumab.mp. not Radiation.mp. not Photodynamic.mp. not Adjuvant.mp. not Neoadjuvant.mp. not Arterial Infusion.mp. [mp=title, abstract, heading word, drug trade name, original title, device manufacturer, drug manufacturer, device trade name, keyword, floating subheading word, candidate term word] |
| **7** | **Google Scholar** | **https://scholar.google.com/** | Cholangiocarcinoma "bile duct cancer" OR "Bile tract cancer" AND "Progression free survival" AND "Overall survival" AND "Clinical Trial" |

**Supplementary Table S2: Results of literature searches**

| **Search** | **Source** | **Date** | **Total results** | **Agreed for retrieval** | **Rejected** | **Duplicates** |
| --- | --- | --- | --- | --- | --- | --- |
| **1** | **Pubmed** | 11 Aug 2021 | 301 | 32 | 265 | 4 |
| **2** | **Pubmed** | 19 Aug 2021 | 722 | 141 | 574 | 7 |
| **3** | **Cochrane Central Register of Controlled Trials (CENTRAL)** | 19 Aug 2021 | 324 | 5 | 173 | 146 |
| **4** | **NIH Database of Clinical Trials** | 20 Aug 2021 | 61 | 2 | 47 | 12 |
| **5** | **World Health Organization International Clinical Trial Registry (WHO-ICTRP)** | 20 Aug 2021 | 319 | 3 | 241 | 75 |
| **6** | **Excerpta Medica database (EMBASE)** | 21 Aug 2021 | 499 | 16 | 232 | 251 |
| **7** | **Google Scholar** | 22 Aug 2021 | 395 | 2 | 324 | 69 |

**Supplementary Table S3:** **Included study’s tumor location and drug dosage information**

| Study | CCA location | Drugs | Dosage | Administration method | Time dose given (days) | Cycle time (days) | Cycles repeated until: |
| --- | --- | --- | --- | --- | --- | --- | --- |
| André  2008 | Extrahepatic/Intrahepatic | Gemcitabine | 1000  mg/m2 | IV | 1 | 14 | Until disease progression, unacceptable toxicity, patient withdrawal of consent, or treatment delay of more than 3 weeks. |
|  |  | Oxaliplatin | 100 mg/m2 | IV | 2 |  |  |
| Lassen 2010 | Extrahepatic/Intrahepatic | Gemcitabine | 600-1,250  mg/m2 | IV | 1, 15 | 28 | The planned treatment duration was six cycles, but treatment could continue at the discretion of the investigator. |
|  |  | Capecitabine | 1000-1,250 mg | Oral | Twice daily |  |  |
|  |  | Oxaliplatin | 60-85  mg/m2 | IV | 1, 15 |  |  |
| Moehler  2014 | Extrahepatic/Intrahepatic | Gemcitabine | 1000 mg/m2 | IV | 1, 8, 15, 22, 29, 36, 43 | 64 | Until disease progression or toxicity |
|  |  | Sorafenib | 400  mg | Oral | Twice daily |  |  |
| Moehler 2014 | Extrahepatic/Intrahepatic | Gemcitabine | 1000 mg/m2 | IV | 1, 8, 15, 22, 29, 36, 43 | 64 | Until disease progression or toxicity |
|  |  | Placebo | 400mg | Oral | Twice daily |  |  |
| Ole Larsen 2015 | Extrahepatic/Intrahepatic/Hilar | Gemcitabine | 1000 mg/m2 | IV | 1 | 14 | Patients continued treatment until progression, or insufficient PFS*, OS**, response rate**, rate converted to resection** and toxicity** |
|  |  | Capecitabine | 650  mg/m2 | Oral |  |  |  |
|  |  | Oxaliplatin | 50 mg/m2 | IV |  |  |  |
| Arima 2017 | Extrahepatic/Intrahepatic | Gemcitabine | 1000 mg/m2 | IV | 1, 8 | 14 | Insufficient response rate*, frequency/severity of toxicities**, PFS**, OS**. |
|  |  | S-1 | 60-100 mg | Oral | 1-14 |  |  |
| Lau 2018 | Extrahepatic/Intrahepatic/Hilar | Everolimus | 5-10mg | Oral | Daily | - | Until disease progression, unacceptable toxicity, investigator discretion, or insufficient disease control rate* at 12 weeks, objective response rate**, PFS**, OS**, or grade 3-4 events** |
| Davis 2018 | N/a | Gemcitabine | 1000 mg/m2 | IV | 1 | 14 | Until disease progression, insufficient objective response*, or a maximum of 12 cycles. |
|  |  | Cisplatin | 35 mg/m2 | IV |  |  |  |
|  |  | 5-flourouracil | 2400 mg/m2 | IV |  |  |  |
| Mazzaferro 2019 | Intrahepatic | Derzatinib | 300-400 mg | Oral | Daily | 28 | Until disease progression, unacceptable toxicity, investigator decision, or consent withdrawal |
| Moehler 2019 | Extrahepatic/Intrahepatic | Gemcitabine | 800-1000 mg/m2 | IV | 1, 8 | 21 | Until disease progression, withdrawal of consent, or maximum of 8 cycles. |
|  |  | Cisplatin | 20-25 mg/m2 | IV | 1, 8 |  |  |
|  |  | Afatinib | 20-40 mg/m2 | Oral | Daily |  |  |
| Belkouz 2020 | Extrahepatic/Intrahepatic/Hilar | Oxaliplatin | 85  mg/m2 | IV | 1 | 14 | Until a maximum of 12 cycles |
|  |  | Leucovorin | 400  mg/m2 | IV |  |  |  |
|  |  | Irinotecan | 180  mg/m2 | IV |  |  |  |
|  |  | 5-fluorouracil | 400  mg/m2 | Bolus |  |  |  |
|  |  | 5-fluorouracil | 2400   mg/m2 | IV |  |  |  |
| Markussen 2020 | Extrahepatic/Intrahepatic | Gemcitabine | 1000  mg/m2 | IV | 1, 8 | 21 | Until disease progression, unmanageable toxicity, or withdrawal of consent |
|  |  | Cisplatin | 25 mg/m2 | IV | 1, 8 |  |  |
| Markussen 2020 | Extrahepatic/Intrahepatic | Gemcitabine | 1000  mg/m2 | IV | 1 | 14 | Until disease progression, unmanageable toxicity, or withdrawal of consent |
|  |  | Capecitabine | 650  mg/m2 | Oral | Twice daily |  |  |
|  |  | Oxaliplatin | 50  mg/m2 | IV | 1 |  |  |
| Ueno 2021 | Extrahepatic/Intrahepatic | S-1 | 80–120 mg | Oral | 1-14 | 21 | Until unacceptable PFS*, OS**, response rate**, Disease control rate**, safety** |
|  |  | Resminostat | 200  mg | Oral | 1-5, 8-12 |  |  |
| Ueno 2021 | Extrahepatic/Intrahepatic | S-1 | 80–120 mg | Oral | 1-14 | 21 | Until unacceptable PFS*, OS**, response rate**, Disease control rate**, safety** |
|  |  | Placebo |  | Oral | 1-5, 8-12 |  |  |
| Zhang 2021 | Extrahepatic/Intrahepatic | Apatinib mesylate | 500  mg | Oral | 1-28 | 28 | Until disease progression |
| Yoo 2021 | Extrahepatic/Intrahepatic | Nal-Irinotecan | 70 mg/m2 | IV | 1 | 14 | Until disease progression |
|  |  | 5-Flourouracil | 2400 mg/m2 | IV |  |  |  |
|  |  | Leucovorin | 400 mg/m2 | IV |  |  |  |
| Yoo 2021 | Extrahepatic/Intrahepatic | 5-Flourouracil | 2400 mg/m2 | IV | 1 | 14 | Until disease progression |
|  |  | Leucovorin | 400 mg/m2 | IV |  |  |  |
| Woodford 2021 | N/a | Capecitabine | 825 mg/m2 | Oral | 1-14 | 21 | Until disease progression |
|  |  | Nab-paclitaxel | 125 mg/m2 | IV | 1, 8 |  |  |

*Primary endpoint

**Secondary endpoint.





**Supplementary Fig. S1:** **Responses in females and males in trials of gemcitabine-based treatment regimens.** (A) Median overall survival (mOS) in months for each of ten treatment groups. (B) Progression-free survival (mPFS) in months for each of nine treatment groups for which data was available.




**Supplementary Fig. S2: Responses in females and males in trials of non-gemcitabine-based treatment regimens.** (A) Median overall survival (mOS) in months for each of nine treatment groups. (B) Progression-free survival (mPFS) in months for each of nine treatment groups.
